# *MAP7* and *MUCL1* Are Biomarkers of Vitamin D3-Induced Tolerogenic Dendritic Cells in Multiple Sclerosis Patients

**DOI:** 10.3389/fimmu.2019.01251

**Published:** 2019-06-19

**Authors:** Juan Navarro-Barriuso, María José Mansilla, Bibiana Quirant-Sánchez, Alicia Ardiaca-Martínez, Aina Teniente-Serra, Silvia Presas-Rodríguez, Anja ten Brinke, Cristina Ramo-Tello, Eva M. Martínez-Cáceres

**Affiliations:** ^1^Division of Immunology, LCMN, Germans Trias i Pujol University Hospital and Research Institute, Barcelona, Spain; ^2^Department of Cellular Biology, Physiology and Immunology, Universitat Autònoma de Barcelona, Bellaterra, Spain; ^3^Multiple Sclerosis Unit, Department of Neurosciences, Germans Trias i Pujol University Hospital, Barcelona, Spain; ^4^Department of Medicine, Universitat Autònoma de Barcelona, Bellaterra, Spain; ^5^Department of Immunopathology, Sanquin Research, and Landsteiner Laboratory, Amsterdam UMC, University of Amsterdam, Amsterdam, Netherlands

**Keywords:** tolerogenic dendritic cells, multiple sclerosis, biomarkers, vitamin D3, immune tolerance

## Abstract

The administration of autologous tolerogenic dendritic cells (tolDC) has become a promising alternative for the treatment of autoimmune diseases, such as multiple sclerosis (MS). Specifically, the use of vitamin D3 for the generation of tolDC (vitD3-tolDC) constitutes one of the most widely studied approaches, as it has evidenced significant immune regulatory properties, both *in vitro* and *in vivo*. In this article, we generated human vitD3-tolDC from monocytes from healthy donors and MS patients, characterized in both cases by a semi-mature phenotype, secretion of IL-10 and inhibition of allogeneic lymphocyte proliferation. Additionally, we studied their transcriptomic profile and selected a number of differentially expressed genes compared to control mature and immature dendritic cells for their analysis. Among them, qPCR results validated *CYP24A1, MAP7* and *MUCL1* genes as biomarkers of vitD3-tolDC in both healthy donors and MS patients. Furthermore, we constructed a network of protein interactions based on the literature, which manifested that *MAP7* and *MUCL1* genes are both closely connected between them and involved in immune-related functions. In conclusion, this study evidences that *MAP7* and *MUCL1* constitute robust and potentially functional biomarkers of the generation of vitD3-tolDC, opening the window for their use as quality controls in clinical trials for MS.

## Introduction

The role of dendritic cells (DC) within the immune system is crucial, since they are in charge of orchestrating immune responses and maintaining the homeostasis between immunogenicity and tolerance. Under normal conditions, DC remain in an immature status (iDC), characterized by their ability to capture and present antigens and other signals in their environment. However, these cells are not stable, and when exposed to a danger signal, iDC become activated and differentiate into professional, antigen-presenting mature DC (mDC). This pro-inflammatory status is characterized by an increase in the expression of MHC and co-stimulatory molecules, thus enabling mDC to induce an efficient and potent immunogenic response (1–4).

If the immune homeostasis is lost, and a breach of tolerance causes mDC to recognize and present specific self-antigens to T cells, different autoimmune pathologies may develop depending on which protein or tissue is targeted, such as multiple sclerosis (MS), rheumatoid arthritis or type 1 diabetes. These complex disorders involve many innate and adaptive mechanisms of the immune system, and their etiology still remains unknown. For this reason, a cure has not been found yet, and the currently available treatments consist in strong immunomodulatory or immunosuppressive drugs. In general, these are focused on addressing the symptoms in a poorly effective and unspecific manner, with potentially severe side effects. Hence, there is an increasing need for new, more specific and effective therapies. Over the last years, tolerogenic DC (tolDC) have been postulated as a novel and promising alternative to treat these disorders (5). In fact, several approaches have already been tested in Phase I clinical trials for autoimmune diseases, as reviewed by ten Brinke et al. (6), and other clinical studies are still ongoing. In all cases, these treatments have demonstrated to be safe, with no relevant side effects on the patients. Consequently, many initiatives are now focused on assessing the actual efficacy of tolerogenic cell therapies.

In general, tolDC are defined as a stable and semi-mature subset of DC with the potential to restore immune tolerance in an antigen-specific manner if loaded with one or more determined peptides, thus not compromising the protective immunity of the patients. Compared to mDC, these cells are typically characterized by their low —or lower— expression of MHC and co-stimulatory molecules (such as CD40, CD80, CD83 or CD86), and by their reduced or null secretion of IL-12, IFN-γ and other pro-inflammatory cytokines, combined with an increment in the production of IL-10 or TGF-β. These features confer to tolDC a reduced capability to induce T cell proliferation and the possibility to prime regulatory T cell (Treg) responses, thus potentially directing the immune response toward a regulatory context (7–9). However, these characteristics can sometimes be very variable, since there is a wide variety of protocols to generate tolDC *in vitro* from human peripheral blood monocytes. These approaches include the use of several compounds, cytokines and immunomodulatory drugs such as IL-10 (10, 11), dexamethasone (11–15) or rapamycin (11, 12, 16), as well as different genetic engineering techniques (17, 18).

Among all of them, the use of 1,25-dihydroxyvitamin D3 —the active form of vitamin D3— to generate tolDC is one of the most widely established protocols. Specifically, vitamin D3-induced tolDC (vitD3-tolDC) present a semi-mature profile, accompanied by an ability to inhibit allogenic T cell proliferation and to polarize the immune response toward an anti-inflammatory T_H_2 profile (12, 19–28). Furthermore, several studies using animal models of autoimmune diseases have demonstrated their functionality *in vivo* (29–31). In general, these cells are characterized by the suppression of the NF-κB pathway (21, 32), accompanied by an increased activity of the oxidative metabolism of glucose, and indeed the glucose availability and the glycolytic activity mediated through mTOR signaling are crucial for the induction and maintenance of their tolerogenic function (27). However, despite the identification of several pathways involved in the anti-inflammatory role of vitD3-tolDC, the specific mechanisms for the induction of immune tolerance by these cells have not been clearly identified yet.

Previously, our group has successfully generated human vitD3-tolDC —demonstrating their tolerogenic properties *in vitro* using cells generated from both healthy donors and MS patient samples—, and has studied their transcriptomic profile compared to other tolDC protocols (12, 25, 26, 33). Additionally, in further *in vivo* studies, we also reported a positive and beneficial effect of antigen-specific vitD3-tolDC treatment over the course of the murine model of MS, the experimental autoimmune encephalomyelitis (30, 31). Altogether, these results have led to the development of an ongoing Phase I clinical trial in MS patients with peptide-loaded vitD3-tolDC (http://www.clinicaltrials.gov, NCT02903537).

However, for the full translation of an autologous, antigen-specific, tolerogenic cell therapy into the common clinical practice, several additional steps yet need to be taken. Among them, the definition of adequate, robust and objective biomarkers constitutes one of the priorities. These markers would, on the one hand, guarantee the proper generation and functionality of tolDC, without compromising the safety for the patients. On the other hand, these biomarkers would enable the comparison of results with other research groups, hereby accelerating the translation of tolDC therapies into the clinic in a collaborative endeavor (6). However, although many efforts have been made in this regard, and despite several genes and molecules have been identified for a variety of tolDC protocols separately, such as *IDO1, GILZ*, or *ANXA1*, the definition of universal biomarkers of tolerance-inducing cell products has not been possible so far, and it seems unlikely provided the wide heterogenicity of approaches being used (34). For this reason, in this study we have analyzed the transcriptomic profile of vitD3-tolDC in order to select and validate several differentially expressed genes (DEG) that may be used as transcriptomic biomarkers of these cells in a clinical trial for MS patients.

## Materials and Methods

### Sample Collection

Buffy coat samples from 24 randomized healthy donors were obtained from the *Banc de Sang i Teixits* (Barcelona, Spain), following the institutional Standard Operating Procedures for blood donation, which included a signed informed consent. Whole blood samples from 10 MS patients were collected by standard venipuncture in lithium heparin tubes. Patients did not receive any corticoid or disease-modifying therapy during at least the previous 2 months, and both relapsing and progressive forms of the disease were considered. The same procedure was followed for whole blood obtention from 34 healthy donors for the functional assays (see below). This study was approved by the Germans Trias i Pujol Hospital ethical committee, and all patients and healthy controls signed an informed consent.

Complementary DNA (cDNA) of paired IL-10-induced tolDC (IL10-tolDC) and mDC samples from 5 healthy donors were obtained from Sanquin Bloodbank (Amsterdam, The Netherlands) after informed consent. These samples were generated as described in Boks et al. (11).

### Monocyte Isolation

Samples from healthy donors were processed first depleting CD3^+^ cells using the RosetteSep® Human Monocyte Enrichment Cocktail kit (StemCell Technologies, Vancouver, Canada) prior to a ficoll-hypaque (Rafer, Zaragoza, Spain) density gradient separation. Subsequently, CD14^+^ cells were isolated by positive selection using the EasySep® Human CD14 Positive Selection Kit (StemCell) following manufacturer's instructions. For the isolation of monocytes from MS patients, peripheral blood monocytes were isolated from 50 mL of whole blood by ficoll-hypaque density gradient separation, followed by the abovementioned CD14 positive selection step. The initial CD3^+^ cells depletion step was not performed due to the limited amount of blood. Samples were acquired on a FACSCanto II flow cytometer (BD Biosciences, Franklin Lakes, NJ, USA), and cell viability was determined using 7-amino-actinomycin D (7-AAD) (BD Biosciences) and phycoerythrin (PE)-conjugated annexin V (Immunotools, Friesoythe, Germany) staining for 20 min at 4°C, protected from light. Cell counts were quantified using PerfectCount microspheres (Cytognos, Salamanca, Spain) and monocyte purity was determined using forward and side scatter gating strategies on FACSDiva software (BD Biosciences).

### VitD3-tolDC Generation

Monocytes from both healthy donors and MS patients were cultured at a density of 1 × 10^6^ cells/mL in 24-well plates at 37°C and a 5% CO_2_ atmosphere for 6 days in 1 mL X-VIVO 15 medium (Lonza, Basel, Switzerland), supplemented with 2% heat-inactivated human AB serum, 2 mM L-glutamine (Sigma-Aldrich, St. Louis, MO, USA), 100 U/mL penicillin (Reig Jofre, Sant Joan Despí, Spain) and 100 μg/mL streptomycin (Normon, Tres Cantos, Spain). For the generation of iDC, monocytes were differentiated in the presence of 200 U/mL granulocyte macrophage colony-stimulating factor (GM-CSF) and 250 U/mL IL-4 (both from Peprotech, London, UK). Whole volume of fresh medium and cytokines was replenished on day 4. In addition, a maturation cocktail containing 1,000 U/mL IL-1β, 1,000 U/mL TNF-α (both from Peprotech) and 1 μM prostaglandin E2 (PGE2) (Pfizer, New York, NY, USA) was added on day 4 to both mDC and vitD3-tolDC conditions. Finally, for the induction of vitD3-tolDC, these cells were besides treated with 1 nM vitamin D3 (Calcijex, Abbott, Chicago, IL, USA) on days 0 and 4. On day 6, all three conditions were harvested following an incubation with accutase (Invitrogen, Carlsbad, CA, USA) for 30 min at 37°C to detach the cells from the plate, and washed twice. Cell counts and viability were determined by flow cytometry, as shown above, and after the phenotypical and functional characterization, dry pellets of each condition were generated by centrifugation and stored at −80°C.

### Phenotype Analysis

Surface protein expression of CD11c, CD14, CD25, CD83, CD86 and HLA-DR of iDC, mDC and vitD3-tolDC was determined by flow cytometry. For each measurement, DC suspensions were incubated for 20 min, protected from light, with the adequate amounts of monoclonal antibodies anti-: CD11c PE-Cyanine dye 7 (PE-Cy7), CD14 Violet 450 (V450), CD25 allophycocyanin (APC), CD83 APC, CD86 fluorescein isothiocyanate (FITC) and HLA-DR Violet 500 (V500) (all of them from BD Biosciences). Afterwards, at least 10,000 CD11c^+^ events of each sample were acquired in a FACSCanto II flow cytometer and analyzed using FACSDiva software.

### Functionality Test

Allogeneic peripheral blood mononuclear cells (PBMC) from whole blood of healthy donors were isolated by ficoll-hypaque density gradient separation. Cells were washed twice afterwards, and their absolute number and viability was determined as shown above.

Subsequently, a proliferation assay was performed in 96-well round bottom plates with co-cultures of 10^5^ allogeneic PBMC and 5,000 iDC, mDC, or vitD3-tolDC (1:20 ratio) in a total volume of 200 μL of supplemented X-VIVO 15 medium. The same number of PBMC cultured in the presence of either supplemented X-VIVO 15 medium or 50 ng/mL phorbol 12-myristate-13-acetate (PMA) and 500 ng/mL ionomycin were used as negative and positive controls, respectively. Six replicates of each condition were performed. Cells were incubated for 4 days at 37°C in a 5% CO_2_ atmosphere.

Finally, 1 μCi [^3^H]-thymidine (PerkinElmer, Waltham, MA, USA) was added to each well and the plate was incubated for further 18 h under the same conditions. Cells were then collected using a HARVESTER96 2M cell harvester (Tomtec Inc, Hamden, CT, USA) and read on a 1450 MicroBeta TriLux liquid scintillation counter (Wallac, Turku, Finland).

### Cytokine and Soluble Protein Production

The production of granzyme B (GZMB) and vascular endothelial growth factor (VEGF), as well as IL-10, IFN-γ and IL-12p70 cytokines, was quantified in the culture supernatants of tolDC using the Human Soluble Protein CBA Flex Set (BD biosciences) according to manufacturer's instructions. Samples were acquired on an LSR Fortessa flow cytometer (BD Biosciences) and analyzed using FACSDiva software.

The production of TGF-β was determined using the Human/Mouse TGF beta 1 Uncoated ELISA kit (Invitrogen) in 100 μL of supernatant samples, again following manufacturer's instructions. The optical density of each well was measured at 450 nm, and the optical density at 570 nm was then substracted as background signal, using a Varioskan Flash Multimode Reader (Thermo Fisher Scientific, Waltham, MA, USA).

### Differential Expressed Genes Selection and Generation of a Network of Protein Interactions

The data of a comparative transcriptomic analysis of vitD3-tolDC, iDC and mDC from 5 healthy donors, previously performed by our group in a microarray study (33), was used to select several DEG. For that, the mean difference of expression (MeanDiff) of each gene was evaluated, and only those genes that were specifically induced or repressed in vitD3-tolDC vs. both iDC and mDC conditions (either MeanDiff _vitD3-*tolDC vs*. *mDC*_ > 0.5 —first criterium—, while MeanDiff _iDC vs. mDC_ < 0.5, —second criterium—; or MeanDiff _vitD3-*tolDC vs*. *mDC*_ < −0.5, while MeanDiff _iDC vs. mDC_ > −0.5), with a statistically significant differential expression in the first criterium (*p* < 0.01), were selected in order to validate them. Unlike in our previous study, B-statistic —an indicator of the likelihood of the results— was not considered for the selection of candidate genes this time. Microarray raw and processed data were deposited in the ArrayExpress database at EMBL-EBI (www.ebi.ac.uk/arrayexpress) under accession number E-MTAB-6937.

Additionally, a bioinformatic analysis was performed in order to characterize the molecular mechanisms involved in the tolerogenicity of vitD3-tolDC. Briefly, the Therapeutic Performance Mapping System technology (35, 36) was used to generate a mathematical model of protein interactions from our transcriptomic microarray data, based on an effectors database of tolDC biology and functionality (Anaxomics, Barcelona, Spain). This database was generated using information available in the literature as well as public and private repositories. From this model, a network of protein interactions between all the effectors found in our microarray data was built using an Artificial Neural Networks analysis and represented using Cytoscape 3.6.1 software.

### RNA Extraction and qPCR Validation

Total RNA was isolated from dry pellets using the RNeasy Mini Kit (Qiagen, Hilden, Germany) along with a complementary DNAse treatment with the RNAse-free DNAse Set (Qiagen), following manufacturer's instructions. Quantity and purity of the samples was then determined using a Nanodrop ND-1000 spectrophotometer (Thermo Fisher Scientific), and the RNA was subsequently retrotranscribed into cDNA using the High Capacity cDNA Reverse Transcription Kit (Applied Biosystems, Foster City, CA, USA). Finally, 250 ng cDNA were preamplified using the TaqMan™ PreAmp Master Mix Kit (Applied Biosystems).

The expression of genes *CA2, CAMP, CLEC5A, CYP24A1, DHRS9, GAPDH, GZMB, IL1R1, MAP7, MUCL1, OS9, PPIA, SNORD30, SPARC, ST6GAL1, TBP* and *THBS1* was determined by quantitative PCR (qPCR) using the respective TaqMan Gene Expression Assays (Applied Biosystems) shown in Supplementary Table 1, following the instructions provided by the manufacturer, in a LightCycler 480 System thermocycler (Roche, Basel, Switzerland). Housekeeping genes *CYPA, TBP* and *GAPDH* were used as controls. The quantitative expression of each gene was calculated based on the 2^−Δ*Cp*^ method (37), using the mean Cp values of the 3 housekeeping genes. The decimal logarithm of fold change (logFC) expression values of each gene were considered for the definition of validation criteria. Similar to the MeanDiff parameter from the microarray, in this case genes were considered as differentially expressed in vitD3-tolDC vs. both iDC and mDC when either logFC _vitD3-*tolDC vs*. *mDC*_ > 0.5 —first criterium—, while logFC _iDC vs. mDC_ < 0.5, —second criterium—; or logFC _vitD3-*tolDC vs*. *mDC*_ < −0.5, while logFC _iDC vs. mDC_ > −0.5, if statistical significance was reached for the first criterium (*p* < 0.05).

### Immunocytochemistry Validation

In order to confirm the qPCR results and analyze the protein expression and distribution of MAP7 and MUCL1 molecules, an indirect immunocytochemistry (ICC) staining was carried out in vitD3-tolDC, mDC and iDC from healthy donors and MS patient samples. Cell culture differentiations of vitD3-tolDC, mDC and iDC were performed in 24-well plates, following the same protocol described above, over 12 mm round coverslips. After 6 days of culture, cells were fixed with 4% paraformaldehyde, permeabilized with 0.5% TWEEN20 (Sigma-Aldrich) and subsequently blocked with 10% goat serum for 15 min. Afterwards, samples were incubated for 1 h at room temperature or overnight at 4°C with the primary antibodies mouse anti-human α-tubulin (Invitrogen) and either rabbit anti-human MAP7 (Invitrogen) or rabbit anti-human MUCL1 (Sigma-Aldrich). Next, cells were washed and later incubated with AlexaFluor (AF) 488 goat anti-mouse IgG and AF 594 goat anti-rabbit IgG secondary antibodies (both from Jackson ImmunoResearch Laboratories Inc, West Grove, PA, USA) for 30 min at room temperature, protected from light. Cells were washed again, and the coverslips were finally mounted using ProLong® Gold Antifade Mountant medium with DAPI (Invitrogen) for nucleus staining. Samples were analyzed on an Axio Observer Z1 fluorescence microscope (Zeiss, Oberkochen, Germany) with a 63x objective, using ZEN software (Zeiss), and the expression of MAP7 and MUCL1 was calculated as the corrected total cell fluorescence (CTCF) of each protein (38, 39) using the FIJI distribution of ImageJ software (40, 41).

### Statistical Analysis

All the statistical analyses were performed with either parametric or non-parametric tests depending on the normality of each compared data set, as determined by the D'Agostino & Pearson test, using Prism 6.0 software (GraphPad, La Jolla, CA, USA). For multiple comparisons, either the one-way ANOVA test with Geisser-Greenhouse correction or the non-parametric Friedman test with Dunn's correction were used depending on the normality of the groups. Analogously, for comparisons between two groups, either the t-Student or the Wilcoxon test were used if the samples were normally distributed or not, respectively. When N ≤ 6, parametric tests were used anyway due to the small sample size (42). Results were expressed as mean ± standard deviation (SD), unless noted otherwise, and they were considered statistically significant when p < 0.05.

## Results

### VitD3-tolDC Show Phenotypical Characteristics of tolDC

TolDC were generated from samples of 24 healthy donors and 10 MS patients with 83.1 ± 0.01% purity of monocytes and 98.4 ± 0.03% viability after CD14^+^ cells positive selection. After *in vitro* differentiation, DC were harvested, and their phenotype, purity and viability were characterized by flow cytometry following the gating strategy depicted in Supplementary Figure 1a. As determined by the percentage of CD11c^+^ cells, purity of DC was always >90%, with 91.1 ± 0.04% viability in both healthy donor and MS patient cells (Supplementary Figure 1b). When we analyzed the phenotype of the cells, the median fluorescence intensity (MFI) values for CD83, CD86 and HLA-DR on each condition were considered. As displayed in Supplementary Figures 1c–e, vitD3-tolDC from healthy donors showed reductions of 83 ± 34%, 59 ± 15% and 71 ± 15% in the expression of CD83, CD86 and HLA-DR, respectively, compared to mDC, and similar to the expression levels showed by iDC. In the case of MS patient-derived vitD3-tolDC, a similar behavior was observed, with reductions of 77 ± 30%, 60 ± 13% and 61 ± 14% in CD83, CD86 and HLA-DR expression, respectively, compared to mDC, and similar to iDC. All the results reached statistical significance for both healthy donors and MS patients (*p* < 0.05).

### VitD3-tolDC Induce Allogeneic Hyporresponsiveness and Produce Anti-inflammatory Cytokines

The functional assay results evidenced that vitD3-tolDC significantly inhibited the proliferation of allogeneic PBMC. As shown in Supplementary Figure 2a, a 73.6 ± 16.6% reduction compared to mDC was observed in healthy donors, similar to the 84.1 ± 10.7% reduction exhibited by iDC. Analogously, although in a less strong degree, for MS patient-derived DC, a 47.9 ± 25.6% and a 46.3 ± 28.9% reduction in the proliferation induction was observed for vitD3-tolDC and iDC, respectively, in comparison to mDC. In all four comparisons, statistical significance was reached (*p* < 0.05).

When the cytokine secretion profile of vitD3-tolDC was compared to mDC and iDC, an increase in the production of the anti-inflammatory cytokines IL-10 (Supplementary Figure 2b) and TGF-β (Supplementary Figure 2c) was detected in vitD3-tolDC differentiated from healthy donors (IL-10 _vitD3-*tolDC*_: 166.0 ± 287.7 pg/mL vs. IL-10 _mDC_: 44.0 ± 51.4 pg/mL; *p* = 0.003; and TGF-β _vitD3-*tolDC*_: 306.5 ± 159.5 pg/mL vs. TGF-β _mDC_: 188.1 ± 165.3 pg/mL; *p* = 0.046), but only of IL-10 in the case of vitD3-tolDC generated from MS patients (IL-10 _vitD3-*tolDC*_: 148.6 ± 141.7 pg/mL vs. IL-10 _mDC_: 62.1 ± 54.1 pg/mL; *p* = 0.043). Furthermore, IL-12 production could not be detected in any condition (data not shown). Finally, no statistically significant changes were found in the production of GZMB (Supplementary Figure 2d), nor IFN-γ (Supplementary Figure 2e), between the different conditions.

### *CYP24A1, MAP7* and *MUCL1* Genes Are Induced in vitD3-tolDC From Healthy Donors and MS Patients

Following the study previously performed by our group in a comparative microarray study of vitD3-tolDC, iDC and mDC from 5 healthy donor samples (33), we applied the filtering criteria described in the methods section to the data results (either MeanDiff _vitD3-*tolDC vs*. *mDC*_ > 0.5 —first criterium—, while MeanDiff _iDC vs. mDC_ < 0.5, —second criterium—; or MeanDiff _vitD3-*tolDC vs*. *mDC*_ < −0.5, while MeanDiff _iDC vs. mDC_ > −0.5, with a statistically significant differential expression in the first criterium). Briefly, these parameters spotted those genes that were specifically induced by the effect of vitamin D3 over DC according to our microarray analysis, since they were differentially expressed in vitD3-tolDC compared to both mDC and iDC, and therefore could be considered as potential transcriptomic biomarkers of vitD3-tolDC (Table 1). As a result, we selected *CA2, CAMP, CLEC5A, CYP24A1, GZMB, IL1R1, MAP7, MUCL1* and *SNORD30* genes for validation.

**Table 1:** Expression by microarray and qPCR of the selected genes in dendritic cells differentiated from healthy donors and multiple sclerosis patients.

**MICROARRAY DATA FROM HEALTHY DONORS [FROM NAVARRO-BARRIUSO ET AL**. **(****33****)]**						
	**iDC vs. mDC**				**vitD3-tolDC vs. mDC**			
**Gene**	***MeanDiff***		***p-value***	***MeanDiff***		***p-value***	
*CA2*	1.044		0.097	1.680		**0.010**	
*CAMP*	0.337		0.279	1.351		**<0.001**	
*CLEC5A*	0.015		0.975	1.573		**0.003**	
*CYP24A1*	−0.585		0.264	2.271		**<0.001**	
*GZMB*	0.097		0.582	0.734		**<0.001**	
*MAP7*	0.075		0.663	0.880		**<0.001**	
*MUCL1*	0.084		0.857	2.132		**<0.001**	
*SNORD30*	−0.040		0.949	1.616		**0.016**	
**HEALTHY DONORS**						
	**iDC vs. mDC**		**vitD3-tolDC vs. mDC**		**vitD3-tolDC vs. iDC**	
**Gene**	***logFC±SD***	***p-value***	***logFC±SD***	***p-value***	***logFC±SD***	***p-value***
*CA2*	0.566 ± 0.391	**0.001**	0.858 ± 0.445	**<0.001**	0.292 ± 0.276	**0.042**
*CAMP*	0.290 ± 0.397	**0.042**	0.957 ± 0.447	**<0.001**	0.668 ± 0.433	**0.003**
*CLEC5A*	−0.254 ± 0.371	0.063	0.602 ± 0.324	**0.001**	0.856 ± 0.346	**<0.001**
*CYP24A1*	−1.680 ± 0.422	**0.008**	1.532 ± 0.637	**0.004**	3.212 ± 0.914	**0.004**
*GZMB*	0.588 ± 0.426	**0.001**	0.607 ± 0.554	**0.001**	0.019 ± 0.515	>0.999
*IL1R1*	−0.133 ± 0.259	0.250	−0.662 ± 0.353	**<0.001**	−0.528 ± 0.293	**<0.001**
*MAP7*	0.473 ± 0.353	**0.002**	1.015 ± 0.220	**<0.001**	0.542 ± 0.308	**0.007**
*MUCL1*	−0.318 ± 0.453	0.130	1.511 ± 0.419	**<0.001**	1.829 ± 0.405	**<0.001**
*SNORD30*	−0.025 ± 0.204	0.056	−0.001 ± 0.207	0.233	0.024 ± 0.278	>0.999
**MULTIPLE SCLEROSIS PATIENTS**						
	**iDC vs. mDC**		**vitD3-tolDC vs. mDC**		**vitD3-tolDC vs. iDC**	
**Gene**	***logFC±SD***	***p-value***	***logFC±SD***	***p-value***	***logFC±SD***	***p-value***
*CAMP*	0.431 ± 0.342	0.042	0.441 ± 0.331	0.076	0.010 ± 0.459	>0.999
*CLEC5A*	−0.837 ± 0.539	**0.002**	0.111 ± 0.249	0.668	0.947 ± 0.490	**0.002**
*CYP24A1*	−0.762 ± 0.739	0.160	1.465 ± 0.908	**<0.001**	2.227 ± 1.448	**<0.001**
*IL1R1*	0.143 ± 0.158	0.103	−0.307 ± 0.179	**0.002**	−0.450 ± 0.214	**0.008**
*MAP7*	0.320 ± 0.296	0.081	0.506 ± 0.297	**0.003**	0.187 ± 0.292	0.174
*MUCL1*	−0.283 ± 1.209	0.221	0.790 ± 0.325	**0.042**	1.073 ± 1.362	**<0.001**

*Gene expression values from a microarray study with healthy donors (n = 5) or from qPCR analysis from healthy donors (n = 24, except for CYP24A1, in which n = 10, and GZMB and SNORD30, in which n = 20) and multiple sclerosis patients (n = 10). Data presented as either the mean difference of expression (MeanDiff) for the microarray data or as the mean decimal logarithm of fold change (logFC) ± SD for the qPCR results. Housekeeping genes GAPDH, TBP and CYPA were used as controls. One qPCR experiment was performed for each donor or patient, with triplicated measurements for each sample. Microarray p-values < 0.01 and qPCR p-values < 0.05 are highlighted in bold. iDC, immature dendritic cells; mDC, mature dendritic cells; vitD3-tolDC, vitamin D3-induced tolerogenic dendritic cells. Friedman test with Dunn's correction or one-way ANOVA test with Geisser-Greenhouse correction*.

**Figure 1:**
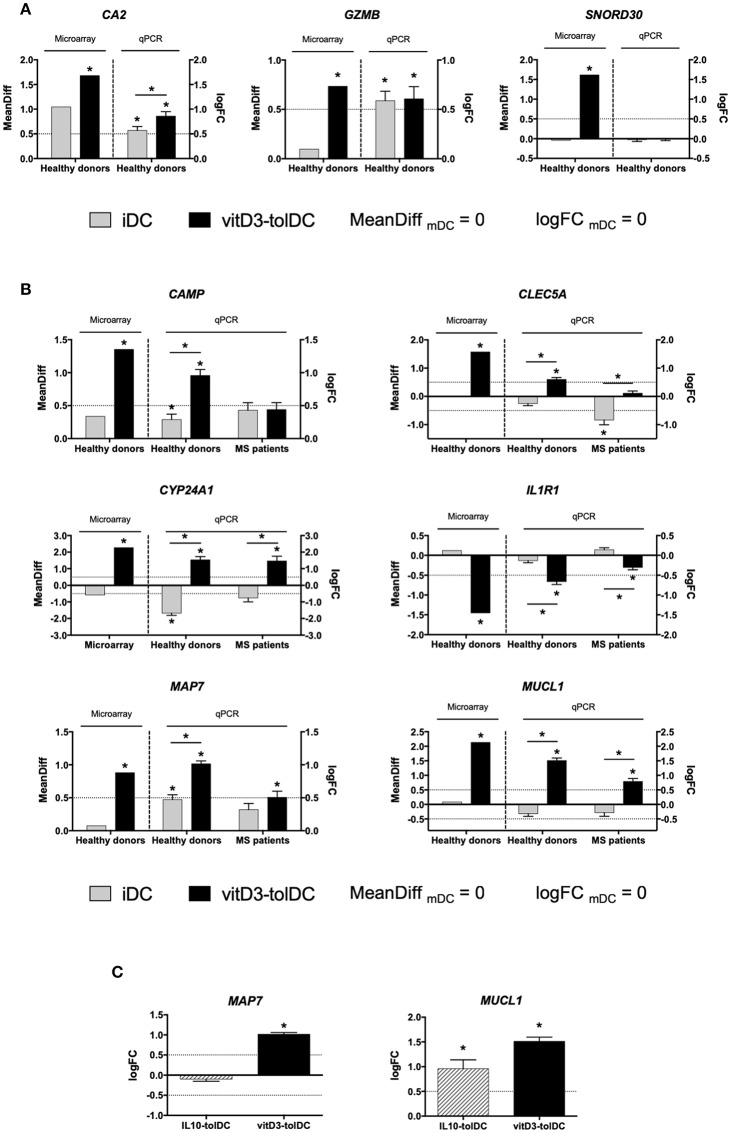
Expression of the selected genes as candidate biomarkers of vitD3-tolDC. **(A)** Expression of *CA2, GZMB* and *SNORD30* in healthy donors by microarray (*n* = 5) and quantitative PCR (qPCR; *n* = 20, except *CA2*, in which *n* = 24) in immature dendritic cells (iDC), mature DC (mDC) and vitD3-tolDC. **(B)** Expression of *CAMP, CLEC5A, CYP24A1, MAP7* and *MUCL1* in healthy donors both by microarray (*n* = 5) and qPCR analysis (*n* = 24, except *CYP24A1*, in which *n* = 10) in iDC, mDC and vitD3-tolDC, and in multiple sclerosis (MS) patients by qPCR only (*n* = 10). **(C)** Expression of *MAP7* and *MUCL1* in vitD3-tolDC (*n* = 24) and in IL10-tolDC (*n* = 5) by qPCR. Data presented as the mean difference of expression (MeanDiff) or the decimal logarithm of fold change (logFC) expression for the microarray and qPCR results, respectively, in both cases normalized to mDC expression. Housekeeping genes *GAPDH, TBP* and *CYPA* were used as controls. One qPCR experiment was performed for each donor or patient, with triplicated measurements for each sample. Error bars corresponding to SEM. Dotted lines represent the logFC = 0.5 or −0.5 expression threshold. *p < 0.05. Friedman test with Dunn's correction, one-way ANOVA test with Geisser-Greenhouse correction or paired *t*-test.

The subsequent qPCR analysis of the actual expression of these genes in healthy donors evidenced that all of them showed an expression pattern compliant with the expression thresholds that were established for the validation —analogously to those from the microarray but depicted in logFC, as defined in the methods section—, except for *CA2* (uncompliant with the second criterium), *GZMB* (uncompliant with the second criterium) and *SNORD30* (uncompliant with the first criterium), as shown in Table 1 and Figure 1A. Consequently, these 3 genes were discarded from further analysis and the expression of the remaining genes was tested on MS patient samples. In this case, Table 1 and Figure 1B show that, however, neither *CAMP*, nor *CLEC5A*, nor *IL1R1* fulfilled our expression criteria. As a result, only *CYP24A1, MAP7* and *MUCL1* could be validated as DEG for our vitD3-tolDC product —reaching statistical significance for the first criterium, as required, in all cases (*p* < 0.05)—, and therefore were selected as transcriptomic biomarkers of our tolDC-inducing protocol.

### *MUCL1* Is Also Induced in tolDC Generated With IL-10

Given the positive results, we therefore intended to test the expression of some of these genes in 5 samples of IL10-tolDC differentiated from healthy donors, in order to assess their potential value as biomarkers of a different tolDC-inducing protocol. While the expression of *CA2, CAMP, CLEC5A, IL1R1* (data not shown) and *MAP7* (Figure 1C) was not altered in these cells, we observed the up-modulation of *MUCL1* gene expression in IL10-tolDC compared to mDC (logFC _IL10-*tolDC vs*. *mDC*_ = 0.960 ± 0.395; *p* = 0.034), similar to that observed for vitD3-tolDC (Figure 1C). The expression of *CYP24A1* was not analyzed, since this gene is directly related to the response of the cells to vitamin D3, which was not present in these specific cultures.

### *MAP7* and *MUCL1* Are Functionally Related in vitD3-tolDC Through a Network of Protein Interactions

**Figure 2:**
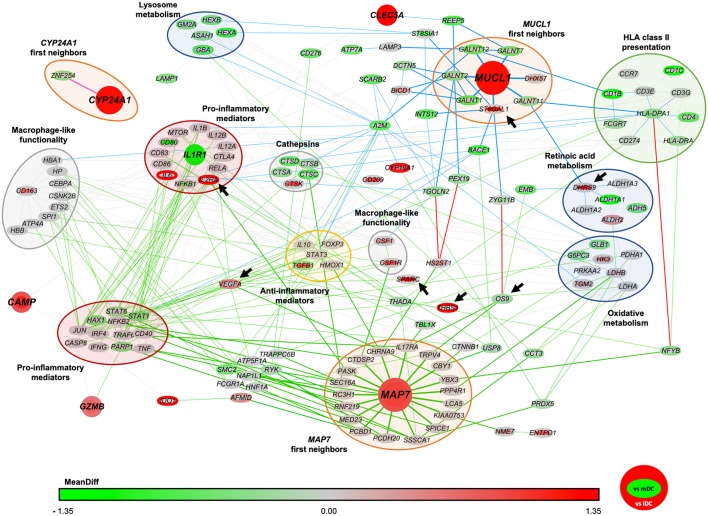
Network of protein interactions between *CYP24A1, MAP7* and *MUCL1*. The protein interactions network was built based in both our microarray results and previously reported data. Each node represents a different protein encoding gene. The color of the border of each node indicates the level of expression of each gene in vitD3-tolDC compared to immature dendritic cells (iDC) and the color of the body of each node indicates the expression of each gene compared to mature dendritic cells (mDC). The color scale indicates the level of mean difference expression (MeanDiff) of each gene according to our microarray study, from green (MeanDiff ≤ −1.35) to red (MeanDiff ≥ 1.35). Green and blue lines indicate protein interactions that are related to *MAP7* and *MUCL1* genes (respectively) in 3 or less parenthood levels, and the red lines indicate interactions that are shared by both genes in <6 parenthood levels. Genes have been grouped in functional clusters, as indicated by the colored bubbles. Arrows indicate those genes that were selected for further validation studies.

In order to find potentially common genes related to *CYP24A1, MAP7* and *MUCL1* that could provide a mechanistic insight in their functionality and the metabolic pathways triggered in vitD3-tolDC, we constructed a network of protein interactions. Briefly, previously reported data from the literature and several public and private databases were crossed with our microarray results in order to link the function of each gene of our transcriptomic study between them. This approach would allow us to reveal potential interactions between our biomarkers that might explain their involvement in immune regulation. Our study revealed that, while *CYP24A1* seemed to be functionally separated from the rest of the genes, *MAP7* and *MUCL1* were closely related through common immune-related mechanisms —such as HLA class II antigen presentation and different anti-inflammatory mediators— and other cellular processes and pathways —highlighting the metabolism of retinoic acid and the oxidative metabolism—, as shown in Figure 2. Furthermore, *CAMP, CLEC5A, GZMB* and *IL1R1* genes appeared closely related to *MAP7* and *MUCL1* in our network.

To validate these results and confirm the close functional relation between *MUCL1* and *MAP7*, we studied the expression of *DHRS9, OS9, SPARC, ST6GAL1* and *THBS1* genes. As shown in Table 2, their expression in our microarray indicated an overexpression of *DHRS9, SPARC* and *ST6GAL* in vitD3-tolDC vs. mDC, but not iDC, while *THBS1* and *OS9* appeared induced and slightly down-modulated, respectively, in vitD3-tolDC compared to iDC. We, therefore, validated their expression in 10 samples from healthy donors and 10 samples from MS patients, and observed that the expression pattern of these 5 genes in healthy donor samples was in accordance to that shown in our microarray data (Table 2). Furthermore, and with the only exception of *DHRS9* and *OS9*, these expression patterns were also confirmed in DC samples obtained from MS patients, as shown in Table 2.

**Table 2:** Expression by microarray and qPCR of the selected genes from the protein interaction network in dendritic cells differentiated from healthy donors and multiple sclerosis patients.

**MICROARRAY DATA FROM HEALTHY DONORS [FROM NAVARRO-BARRIUSO ET Al.(****33****)]**				
	**vitD3-tolDC vs. mDC**		**vitD3-tolDC vs. iDC**	
**Gene**	***MeanDiff***	***p-value***	***MeanDiff***	***p-value***
*DHRS9*	1.160	**0.007**	−0.161	0.685
*IL2RA*	−1.539	0.040	2.297	**0.003**
*OS9*	−0.014	0.926	−0.478	**0.004**
*SPARC*	1.347	**<0.001**	0.506	0.101
*ST6GAL1*	1.564	**<0.001**	0.189	0.570
*THBS1*	−1.052	0.048	1.944	**<0.001**
*VEGFA*	0.200	0.509	0.696	0.028
**qPCR DATA FROM HEALTHY DONORS**				
	**vitD3-tolDC vs. mDC**		**vitD3-tolDC vs. iDC**	
**Gene**	***logFC±SD***	***p-value***	***logFC±SD***	***p-value***
*DHRS9*	0.683 ± 0.387	**0.002**	n/a	
*OS9*	−0.011 ± 0.172	0.917	−0.265 ± 0.182	**0.015**
*SPARC*	1.210 ± 0.346	**0.003**	n/a	
*ST6GAL1*	0.571 ± 0.223	**0.002**	n/a	
*THBS1*	−0.023 ± 0.285	0.694	2.508 ± 0.303	**0.004**
**qPCR DATA FROM MULTIPLE SCLEROSIS PATIENTS**				
	**vitD3-tolDC vs. mDC**		**vitD3-tolDC vs. iDC**	
**Gene**	***logFC±SD***	***p-value***	***logFC±SD***	***p-value***
*DHRS9*	0.264 ± 0.358	0.557	n/a	
*OS9*	−0.036 ± 0.152	>0.999	−0.190 ± 0.222	0.076
*SPARC*	0.552 ± 0.194	**0.011**	n/a	
*ST6GAL1*	0.403 ± 0.331	**0.001**	n/a	
*THBS1*	−0.027 ± 0.216	0.420	2.391 ± 0.381	**0.002**

*Gene expression values from a microarray study with healthy donors (n = 5) or from qPCR analysis from healthy donors (n = 10) and multiple sclerosis patients (n = 10). Data presented as either the mean difference of expression (MeanDiff) for the microarray data or as the mean decimal logarithm of fold change (logFC) ± SD for the qPCR results. Housekeeping genes GAPDH, TBP and CYPA were used as controls for the qPCR experiments. One qPCR experiment was performed for each donor or patient, with triplicated measurements for each sample. Microarray p-values < 0.01 and qPCR p-values < 0.05 are highlighted in bold. iDC, immature dendritic cells; mDC, mature dendritic cells; vitD3-tolDC, vitamin D3-induced tolerogenic dendritic cells. Friedman test with Dunn's correction, one-way ANOVA test with Geisser-Greenhouse correction, Wilcoxon matched-pairs signed rank test or paired t-test*.

Additionally, *VEGFA* and *IL2RA* were also studied due to their relevance in our network, but their expression was validated at the protein level. Their gene expression in our microarray study is shown in Table 2. VEGF production (encoded by the *VEGFA* gene) was analyzed by cytometric bead array (CBA) in supernatant samples from 10 healthy donors and 8 MS patients, and the surface expression of CD25 (encoded by the *IL2RA* gene) was assessed by flow cytometry in 6 healthy donor samples. First, in accordance with the results from the microarray (Table 2), an increase in the production of VEGF was evidenced in vitD3-tolDC from healthy donors compared to iDC —since it could not be detected in this condition—, but no statistical significance could be reached in the reduction found compared to mDC (VEGF _vitD3-tolDC_: 205.5 ± 276.9 pg/mL; VEGF _mDC_: 422.2 ± 497.6 pg/mL). Furthermore, similar results were evidenced in samples from MS patients, and VEGF production could not be detected on iDC either (VEGF _vitD3-*tolDC*_: 331.8 ± 321.5 pg/mL; VEGF _mDC_: 369.2 ± 243.1 pg/mL). On the other hand, the study of the expression of CD25 in healthy donor samples from the microarray evidenced a strong up-modulation of the *IL2RA* gene in vitD3-tolDC compared to iDC only. The expression of this gene in vitD3-tolDC compared to mDC, however, did not reach statistical significance (Table 2). Accordingly, our results in samples from 6 healthy donor DC cultures showed a statistically significant increase (p = 0.040) in the MFI values of this marker in vitD3-tolDC compared to iDC, but not mDC, probably due to the high variability observed in this specific condition (CD25 _vitD3-tolDC_: 433.8 ± 194.9; CD25 _mDC_: 796.3 ± 621.2; CD25 _iDC_: 175.0 ± 16.8).

### Protein Expression of Both *MAP7* and *MUCL1* Is Strongly Induced in vitD3-tolDC

**Figure 3:**
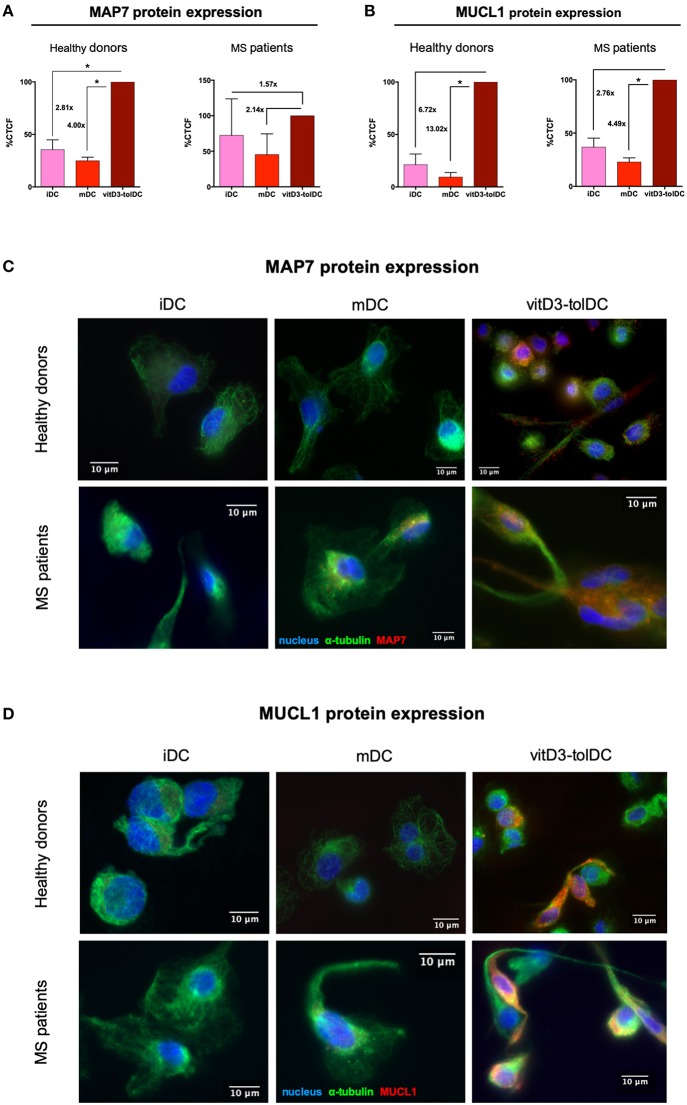
Immunocytochemistry study of MAP7 and MUCL1 protein expression in dendritic cells. **(A)** Relative levels of expression of microtubule-associated protein 7 (MAP7) in dendritic cells differentiated from healthy donor samples (*n* = 4) and MS patient samples (*n* = 3). **(B)** Relative levels of expression of mucin-like 1 (MUCL1) in dendritic cells differentiated from healthy donor samples (*n* = 4) and MS patient samples (*n* = 3). Results are calculated as percentage (%) of corrected total cell fluorescence (CTCF) in immature dendritic cells (iDC) and mature DC (mDC) compared to vitD3-tolDC. One single immunocytochemistry experiment was performed for each sample. Error bars corresponding to SEM. *p < 0.05. One-way ANOVA test with Geisser-Greenhouse correction or paired *t*-test. Representative pictures of the expression of **(C)** MAP7 and **(D)** MUCL1 in iDC, mDC and vitD3-tolDC from healthy donor and MS patient samples. α-tubulin staining is shown in green; either MAP7 or MUCL1 staining is shown in red; nuclei staining is shown in blue. The immunocytochemistry analysis was performed on a fluorescence microscope using a 63x objective. Immunocytochemistry primary staining was performed using mouse anti-human α-tubulin and either rabbit anti-human MAP7 or rabbit anti-human MUCL1 antibodies. Secondary stainings were performed using AlexaFluor (AF) 488 goat anti-mouse IgG and AF 594 goat anti-rabbit IgG antibodies. Nuclei staining was performed using DAPI.

Since *MAP7* and *MUCL1* proved themselves as transcriptomic biomarkers of vitD3-tolDC in both healthy donors and MS patient samples and, additionally, they were closely related between them in our functional network, we further analyzed them and determined the actual expression of their respective encoded proteins in order to provide more reliability to our qPCR results. The ICC analysis in 4 healthy donor samples evidenced that, in fact, a strong up-modulation of these proteins was observed in vitD3-tolDC compared to both iDC and mDC conditions. As shown in Figure 3A, the microtubule-associated protein 7, encoded by *MAP7*, showed a 2.81-fold and a 4.00-fold higher CTCF expression in vitD3-tolDC compared to iDC and mDC, respectively (MAP7 _vitD3-*tolDC*_ = 198.35 ± 40.45 × 10^3^; MAP7 _mDC_ = 49.58 ± 17.16 × 10^3^; MAP7 _iDC_ = 70.54 ± 36.39 × 10^3^), reaching statistical significance in both cases (*p* = 0.016; *p* = 0.007, respectively). The induction of the *MUCL1*-encoded protein was even stronger, as evidenced by the 6.72-fold and 13.02-fold CTCF expression in vitD3-tolDC vs. iDC and mDC, respectively (MUCL1 _vitD3-*tolDC*_ = 1204.85 ± 509.91 × 10^3^; MUCL1 _mDC_ = 92.54 ± 94.19 × 10^3^; MUCL1 _iDC_ = 179.19 ± 32.93 × 10^3^). In this case, however, statistical significance was only reached in the comparison vs. mDC (*p* = 0.024), probably due to the small sample size (Figure 3B). Representative microscopy pictures of the expression of both proteins in healthy donor samples are shown in Figures 3C,D. Furthermore, the ICC analysis in 3 MS patient samples presented a similar tendency. On the one hand, as shown in Figure 3A, the study of MAP7 expression presented a 1.57-fold and a 2.14-fold higher CTCF expression in vitD3-tolDC compared to iDC and mDC, respectively (MAP7 _vitD3-*tolDC*_ = 132.68 ± 91.61 × 10^3^; MAP7 _mDC_ = 61.74 ± 31.52 × 10^3^; MAP7 _iDC_ = 84.32 ± 66.48 × 10^3^). On the other hand, and as shown in Figure 3B, the expression of MUCL1 was, again, even stronger, with a 2.76-fold and 4.49-fold CTCF expression in vitD3-tolDC compared to iDC and mDC, respectively (MUCL1 _vitD3-*tolDC*_ = 132.34 ± 46.44 × 10^3^; MUCL1 _mDC_ = 29.47 ± 11.66 × 10^3^; MUCL1 _iDC_ = 47.97 ± 24.65 × 10^3^). However, statistical significance for MS patient samples could only be reached for the expression of MUCL1 in vitD3-tolDC compared to mDC (*p* = 0.049), again, probably due to the small sample size. Representative microscopy pictures of the expression of both proteins in MS patient samples are shown in Figures 3C,D.

## Discussion

The identification of biomarkers is a key point for the translation of tolDC into the clinic. In this article, we have evidenced that *CYP24A1, MAP7* and *MUCL1* genes appear strongly induced in vitD3-tolDC, both in healthy donors and MS patients. Therefore, the differential expression of these genes in our tolerogenic cell product gives them the potential to unequivocally identify vitD3-tolDC with a simple qPCR analysis, without ambiguity, thus ensuring that they are not immunogenic (not mDC), nor susceptible of maturation (not iDC), and consequently characterizing their tolerogenic potential. Additionally, the study of the protein expression of *MAP7* and *MUCL1* further supported these results. The study of these genes in the context of the whole transcriptome of the vitD3-tolDC has also elucidated that the role of *MAP7* and *MUCL1* —but not of *CYP24A1*— seems to be closely related with important and widely described immune- and non-immune-related pathways, which correlates with many of the results that we have obtained at the phenotypical (reduction of co-stimulatory molecules and HLA-DR expression), functional (increased secretion of IL-10 and TGF-β; non detectable production of IL-12) and transcriptomic levels (reduction of *IL1R1* gene expression). Furthermore, many of the interactions suggested by our network have also been confirmed in both healthy donor and MS patient cells. Therefore, even though the specific role of *MAP7* and *MUCL1* in the tolerogenic functionalities of vitD3-tolDC is not fully clear, our results manifested that they are at least having an effect on several relevant immune regulatory mechanisms and different metabolic pathways. On the other hand, *CYP24A1* gene, encoding the vitamin D3 24-hydroxylase, might well serve as a strong and robust biomarker of vitD3-tolDC, although it seems to have little to no influence in the actual regulatory properties of these cells. After all, this gene is directly involved in the metabolism of vitamin D3 (43, 44), and thus could constitute a robust indicator of the response of the cell product to the treatment with this compound. Even though we previously suggested the potential of *CYP24A1, MAP7* and *MUCL1* as candidate biomarkers of vitD3-tolDC in a previous microarray study (33), the current report constitutes their first validation as such.

Several years ago, mucin-like 1, the protein encoded by *MUCL1* gene, was initially identified as a breast-specific gene expressed in more than 90% of breast cancer cell lines, developing an important role in the proliferation of these tumor cells (45–48). However, no other specific role has been reported for it. In fact, its name comes given by the structural analogy of MUCL1 protein with mucin proteins, characterized by regions of high tandem repeated serine and threonine content with extensive O-glycosylation of these residues (46). On the other hand, we have *MAP7*. This gene encodes the microtubule-associated protein 7, a cytoskeleton component that has been mainly related with the development of collateral axon morphogenesis and development in neurons (49, 50). For both MUCL1 and MAP7, a potential role in DC function has not been reported yet. However, interestingly, microtubule associated proteins have been described to be O-glycosylated —just like MUCL1—, although the functional significance of this modification remains to be determined (51). In the case of vitD3-tolDC, our results have demonstrated a strong induction of gene and protein expression of both *MUCL1* and *MAP7*, as already hinted in our previous microarray study, thus suggesting that they might be developing an important role in the functionality of these cells. However, further functional studies should be performed first in order to elucidate to what extent these genes might be involved in the mechanisms of immune tolerance. In any case, the fact that our results also evidence a strong induction of *MUCL1* in IL10-tolDC indicates that this gene might constitute a potential biomarker of the regulatory function of tolDC, and it would be interesting to test its expression in other protocols generating tolerance-inducing cell products. Nevertheless, on the other hand, it is also true that our above-mentioned preliminary microarray results did not show the overexpression of *MUCL1* in dexamethasone nor rapamycin-induced tolDC, although proper PCR validations should be performed in order to confirm this.

In concordance with previous reported studies by our own group (26), and with the objective to finally develop an autologous tolerogenic cell-therapy for MS, our results demonstrated that the generation of vitD3-tolDC in both healthy donors and MS patients did not significantly differ regarding their phenotypic and functional characteristics —with the exception of an increase on TGF-β production in healthy donor samples only—. In all cases, a consistent decrease in the surface expression of CD86 and HLA-DR, as well as in the induction of allogeneic proliferation, was observed, accompanied by a significant increase in the production of IL-10 and no *IL-12* detection. However, the tolerogenic potential of vitD3-tolDC generated from monocytes of MS patients, although sufficient, did not seem as robust as in healthy donors, provided that the changes in their functional and phenotypic markers were not as pronounced. Interestingly, though, the study of *CYP24A1* expression raised another concern in this regard. Even though this gene constitutes a robust biomarker of the generation of vitD3-tolDC, as discussed above, its expression also manifested important differences in iDC from healthy donors and MS patients; compared to mDC, a strong repression of *CYP24A1* was observed in healthy donor samples, but the expression of this gene in iDC from MS patients was similar to mDC. Therefore, this result implicates that the transcriptomic profile of DC in MS patients is already different before receiving the same treatment with vitamin D3, thus suggesting a more pro-inflammatory baseline status in these cells. However, the strong induction of *CYP24A1* observed in vitD3-tolDC still indicates that these cells are adequately responding to the effect of vitamin D3, suggesting that the problem has to be downstream.

These differences between healthy donors and MS patient samples also forced us to discard another interesting candidate transcriptomic biomarker, *DHRS9*, the gene encoding the dehydrogenase/reductase 9, involved in the metabolism of retinoic acid (52). The relevance of this gene comes given by its potential as a broad-use biomarker of immune tolerance, since it has been already described as differentially induced in vitamin D3 + dexamethasone-induced tolDC (53) and, especially, in regulatory macrophages (54). It is also worth noting that, even though *DHRS9* could not be confirmed as a biomarker of vitD3-tolDC from MS patients, this does not mean that it could not still be useful in cells generated from patients with a different autoimmune disease, such as rheumatoid arthritis or type 1 diabetes. In this regard, our results already allowed us to point out some transcriptomic differences between healthy donors and MS patients. We observed that *CLEC5A* could not be validated in MS patient samples either, and when we studied our network, we noticed that both *CLEC5A* and *DHRS9* were closely related to *MUCL1* through *GALNT2, GALNT7* and *GALNT11* genes. Specifically, these 3 genes have been described to be in charge of the processes of O-linked glycosylation. Consequently, this might be an indicator of a potential misfunction in the glycosylation mechanisms of MS patients, that might be indirectly affecting the expression of *CLEC5A, DHRS9* and probably many other genes. In fact, several glycosylation defects have already been related with the pathogenesis of MS, as reviewed by Grigorian et al. (55). However, *MUCL1* gene expression would also be expected to be affected by a defect in these genes, but our results showed that this was not the case. As a possible explanation, the glycosylation of *MUCL1* might be sufficiently mediated by *ST6GAL1* gene alone —whose expression still remains slightly induced in vitD3-tolDC from MS patient samples—. In any case, our results have raised the need to explore the mechanisms that might be different between healthy donors and MS patients. On the other hand, most of the genes closely related to *MAP7* showed a similar behavior in both healthy donor and MS patient vitD3-tolDC, such as *SPARC, THBS1* or, specially, *VEGFA*, which has been reported to develop a role in the recruitment, inhibition of maturation and IDO1 induction in DC (56–58).

In conclusion, our results evidenced that, despite not having an obvious involvement in the tolerogenic functionality of the cells in all cases, several genes have shown a strong differential expression in vitD3-tolDC from healthy donors, compared to both mature and immature control conditions. Among them, *CYP24A1, MAP7* and *MUCL1* have also been validated as robust transcriptomic biomarkers of vitD3-tolDC generated from MS patient samples. Thus, this finding opens a promising window for the use of these genes as a reliable quality control in clinical trials with a simple qPCR analysis, before administering the cell product into the patients. Furthermore, the role of *MAP7* and *MUCL1*, but not of *CYP24A1*, seems to be strongly related to important immune-related functions. Specifically, the case of *MUCL1* is of significant relevance, since this gene has also demonstrated an interesting potential as a broad-use biomarker of tolerance, based on its validation both in vitD3-tolDC and in IL10-tolDC. Consequently, *MUCL1* sets an interesting path for future experiments with the objective to validate the role of this gene as a potential biomarker of other tolDC protocols, and thus, hopefully as a wide biomarker of tolerogenic cell products and their mechanisms of immune tolerance induction.

## Ethics Statement

This study was approved by the Germans Trias i Pujol Hospital ethical committee, and all patients and healthy controls signed an informed consent.

## Author Contributions

EM-C, JN-B, and MM conceived the experiments. CR-T and SP-R obtained the patient samples. AtB provided cDNA samples from IL10-tolDC. AA-M, BQ-S, JN-B, and MM performed the cell cultures. AA-M, BQ-S, JN-B, and MM performed the cell characterization analyses. JN-B and MM analyzed the results. EM-C, JN-B, and MM interpreted the results. JN-B wrote the manuscript. AA-M, AtB, AT-S, BQ-S, CR-T, EM-C, JN-B, MM, and SP-R reviewed the manuscript.

### Conflict of Interest Statement

The authors declare that the research was conducted in the absence of any commercial or financial relationships that could be construed as a potential conflict of interest.
