# Supplementary material for: MAP7 and MUCL1 Are Biomarkers of Vitamin D3-Induced Tolerogenic Dendritic Cells in Multiple Sclerosis Patients
Source: Front Immunol. 2019 Jun 19;10:1251. doi: 10.3389/fimmu.2019.01251 (PMC6598738; doi:10.3389/fimmu.2019.01251)
Supplement: Supplementary file 1 [file Table_1.pdf]

SUPPLEMENTARY TABLE 1 l TaqMan Assays used for qPCR validation.

Gene	TaqMan Assay ID	Amplicon length (bp)
CA2	Hs01070108_m1	77
CAMP	Hs00189038_m1	86
CLEC5A	Hs00183780_m1	65
CYP24A1	Hs00167999_m1	123
DHRS9	Hs00608375_m1	66
GAPDH	Hs99999905_m1	122
GZMB	Hs01554355_m1	134
IL1R1	Hs00991002_m1	152
MAP7	Hs01009609_m1	91
MUCL1	Hs00536495_m1	86
OS9	Hs00907099_g1	68
PPIA	Hs99999904_m1	98
SNORD30	Hs03309259_s1	70
SPARC	Hs00234160_m1	76
ST6GAL1	Hs00949382_m1	120
TBP	Hs99999910_m1	127
THBS1	Hs00962908_m1	59
