# Supplementary figures and images for: MAP7 and MUCL1 Are Biomarkers of Vitamin D3-Induced Tolerogenic Dendritic Cells in Multiple Sclerosis Patients
Source: Front Immunol. 2019 Jun 19;10:1251. doi: 10.3389/fimmu.2019.01251 (PMC6598738; doi:10.3389/fimmu.2019.01251)

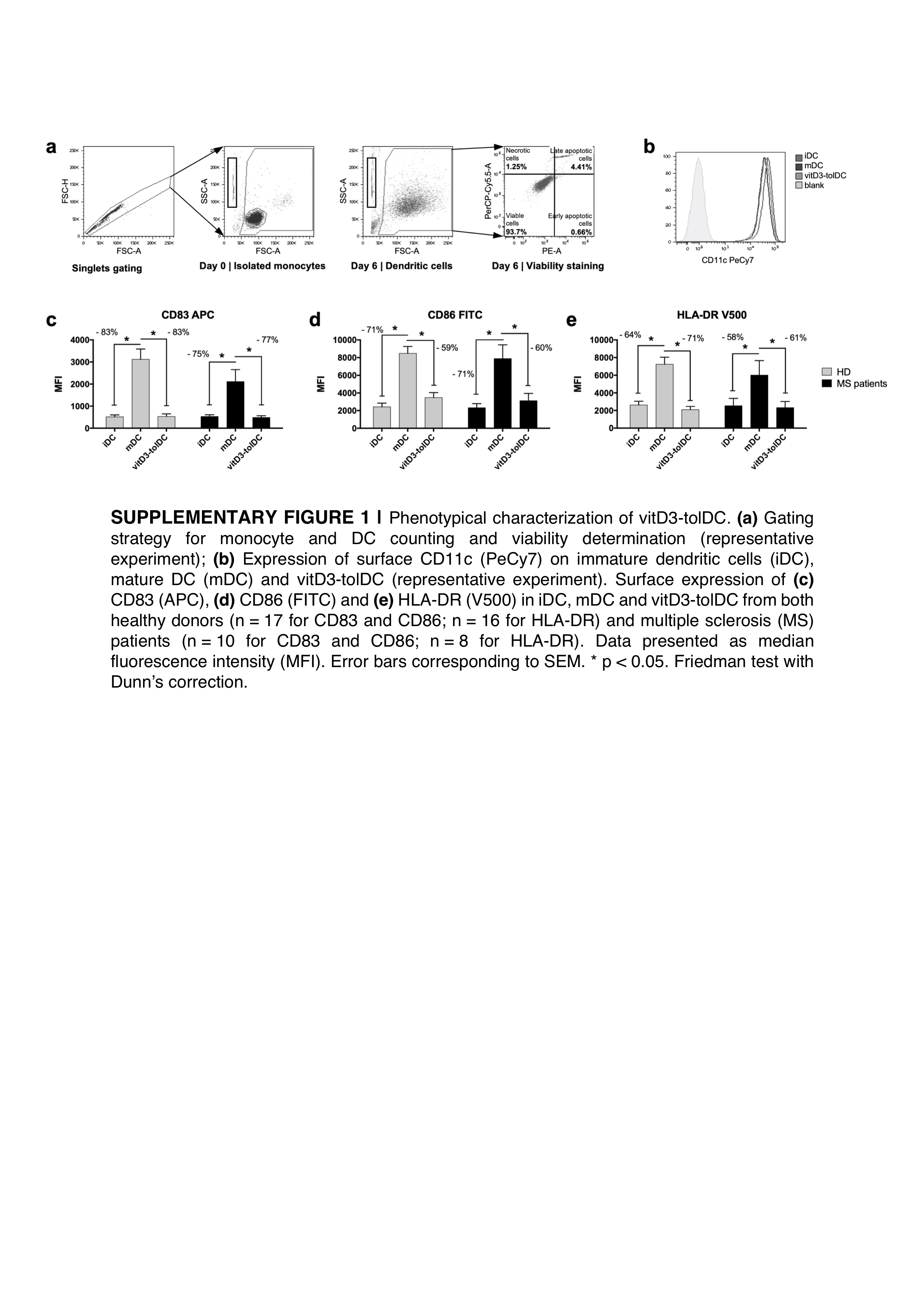

Supplement: Supplementary file 2 [file Image_1.jpg]

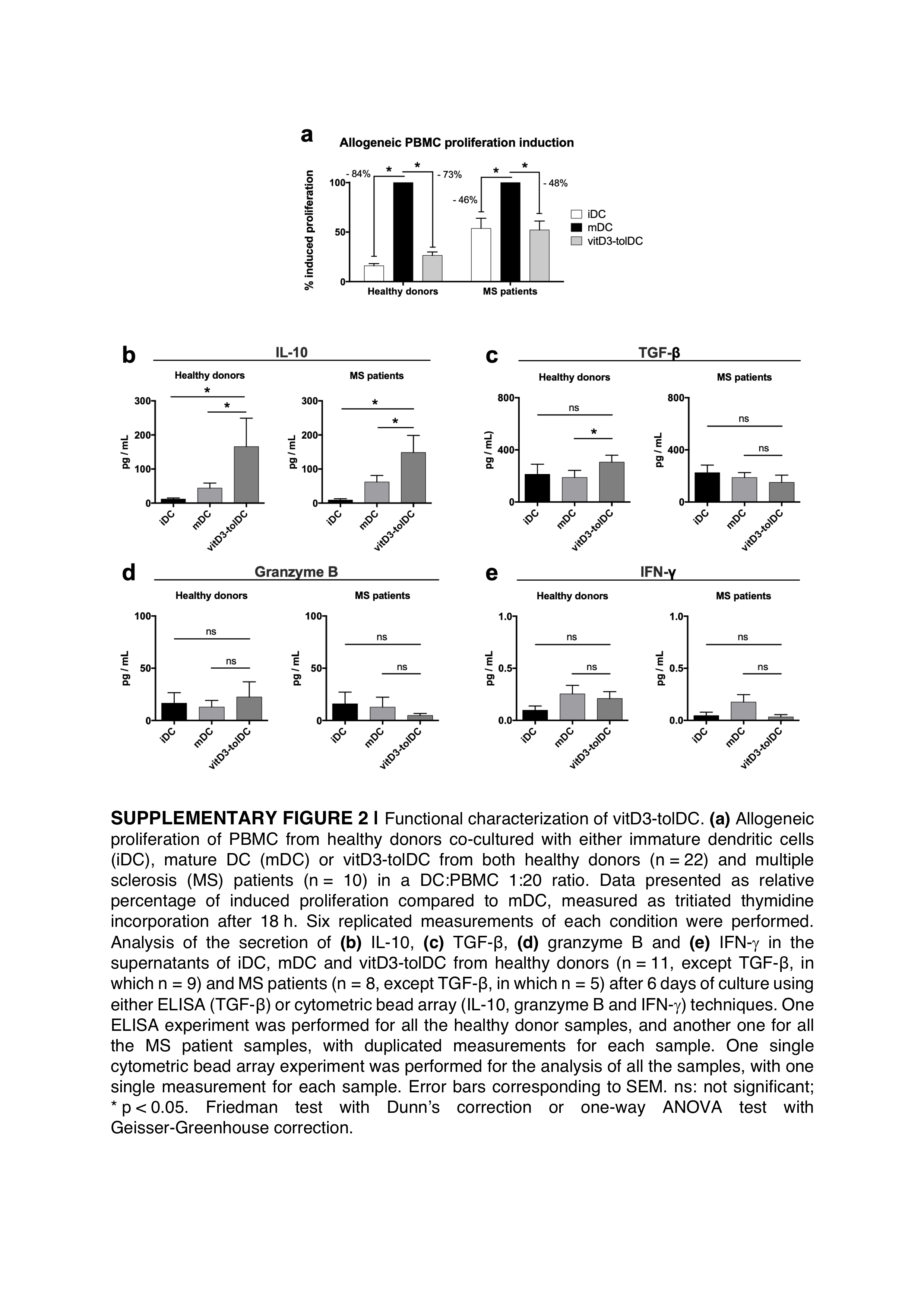

Supplement: Supplementary file 3 [file Image_2.jpg]
